# Electrochemotherapy in the treatment of maxillary multilobular bone tumor in a dog: clinical, histopathological, and tomographic findings

**DOI:** 10.29374/2527-2179.bjvm011225

**Published:** 2026-03-04

**Authors:** Priscila Oliveira Martins, Guilherme Freitas de Souza, André Gustavo Alves Holanda, Denner Santos dos Anjos

**Affiliations:** 1 Self-employed veterinarian, Patas e Focinhos Veterinary Clinic, Mogi Guaçu, SP, Brazil.; 2 Departamento de Cirurgia, Escola de Medicina Veterinária e Ciência Animal, Universidade de São Paulo, São Paulo, SP, Brazil.; 3 Departamento de Cirurgia Veterinária e Reprodução Animal, Estadual Paulista (UNESP), Botucatu, SP, Brazil.

**Keywords:** elechtrochemotherapy, multilobular bone tumor, oral tumor, eletroquimioterapia, tumor ósseo multilobular, tumor oral

## Abstract

Multilobular Bone Tumor (MBT) is a rare, slow-growing, and locally invasive neoplasm in middle-aged to older dogs, occurring predominantly in flat bones of the skull, pelvis, or ribs. This work reports the case of a 10-year-old, castrated, male Schnauzer, presenting with a tumorous growth in the incisor region measuring 1.0 x 0.7 x 0.6 centimeters for 30 days. Excisional biopsy revealed polygonal, hyperchromatic mesenchymal cells with oval nuclei and condensed chromatin, confirming a well-differentiated MLTB. However, the tumor recurred within 5 months, measuring 3.3 x 2.4 centimeters in the same region. Computed tomography (CT) revealed a mixed bone reaction, predominantly lytic, involving the incisive bone at the level of the incisor and canine teeth. The owner declined rostral maxillectomy and opted for a palliative approach with electrochemotherapy (bleomycin 15,000 UI/m^2^) at the tumor site. Complete remission was achieved within 28 days with macroscopic areas suggestive of scarring. The disease-free interval was 365 days without adjuvant therapy. However, at 435 days post-procedure (D0), recurrence was observed in the incisor region, accompanied by respiratory distress. Staging revealed pulmonary metastasis, leading to a decision for humane euthanasia owing to poor prognosis. Electrochemotherapy for oral MLTB in this dog demonstrated good efficacy, prolonged local control, and no regional functional impairments.

## Resumo

O Tumor Ósseo Multilobular (MLTB) é um tumor raro, de crescimento lento e localmente invasivo em cães de meia idade a idosos, ocorrendo principalmente em ossos chatos do crânio , pelve ou costela.. Esse trabalho relata um macho, castrado, raça Schnauzer, de 10 anos de idade, com crescimento tumoral na região dos incisivos de 1,0 x 0,7 x 0,6 centímetros há 30 dias. A biópsia excisional foi realizada evidenciando células mesenquimais poligonais, hipercromática, núcleo oval, cromatina condensada confirmando MLTB bem diferenciado. Entretanto, houve recidiva em 5 meses de 3,3 x 2,4 centímetros na mesma região. A tomografia computadorizada evidenciou reação óssea mista, predominantemente lítica, envolvendo osso incisivo ao nível de dentes incisivos e caninos. A maxilectomia rostral foi recusada pelo tutor, optando pela abordagem paliativa com eletroquimioterapia (bleomicina 15,000 UI/m^2^) na região tumoral. Em 28 dias houve remissão completa com áreas macroscópicas sugestivas de tecido cicatricial. O tempo livre de doença foi de 365 dias sem terapia adjuvante. Entretanto, aos 435 dias do procedimento (D0), houve recidiva na região dos incisivos com alteração respiratória. No estadiamento, foi observada metástase pulmonar sendo optado pela eutanásia humanitária devido ao prognóstico ruim. O uso da EQT para MLTB oral neste cão mostrou boa eficácia, controle local prolongado e sem alteração funcional da região.

## Introduction

Multilobular tumors of the bone (MLTB), also known as multilobular osteosarcomas, generally exhibit slow expansive growth and metastatic potential. While they primarily affect the canine species, cases have also been reported in cats and other species (Murphy et al., 2020). These tumors typically occur in middle-aged to older dogs of medium to large breeds, with no established breed or sex predilection (Marangon et al., 2020; Thompson & Dittmer, 2017).

MLTB typically affects the flat bones of the skull, most frequently involving the maxilla, mandible, orbit, tympanic bulla, and hard palate (Fontes et al., 2023). Although historically identified by various terms, such as multilobular osteochondrosarcoma, chondroma rodens, or juvenile aponeurotic fibroma (Leonardi et al., 2014), the name multilobular tumor of bone (MLTB) is now the preferred designation for both benign and malignant variants (Thompson & Dittmer, 2017).

Clinical manifestations of MLTB depend on the lesion's anatomical location and the tumor's aggressiveness. These may include malocclusion, trismus, and ocular signs such as exophthalmos, as well as neurological deficits and facial disfigurement. Initially, the tumor compresses adjacent structures, leading to soft tissue atrophy. However, over time, MLTB becomes locally infiltrative and develops metastatic potential, frequently disseminating to the lungs and other organs (Fontes et al., 2023).

Metastatic lesions in MLTB often evolve slowly and progressively, allowing patients to remain asymptomatic for over a year. Research by Dernell et al. (1998) indicates high rates of treatment failure, with 47% of surgically treated dogs experiencing local recurrence and 56% developing metastasis. For patients undergoing surgery, the median time to local recurrence was 797 days. Despite these risks, local excision with histologically clean margins can lead to favorable outcomes and prolonged disease control (Banks & Straw, 2004).

This study aims to report the use of electrochemotherapy (ECT) as an alternative treatment for a multilobular tumor of bone (MLTB) located in the rostral maxillary region of a Schnauzer dog.

## Case description

**Figure 1:**
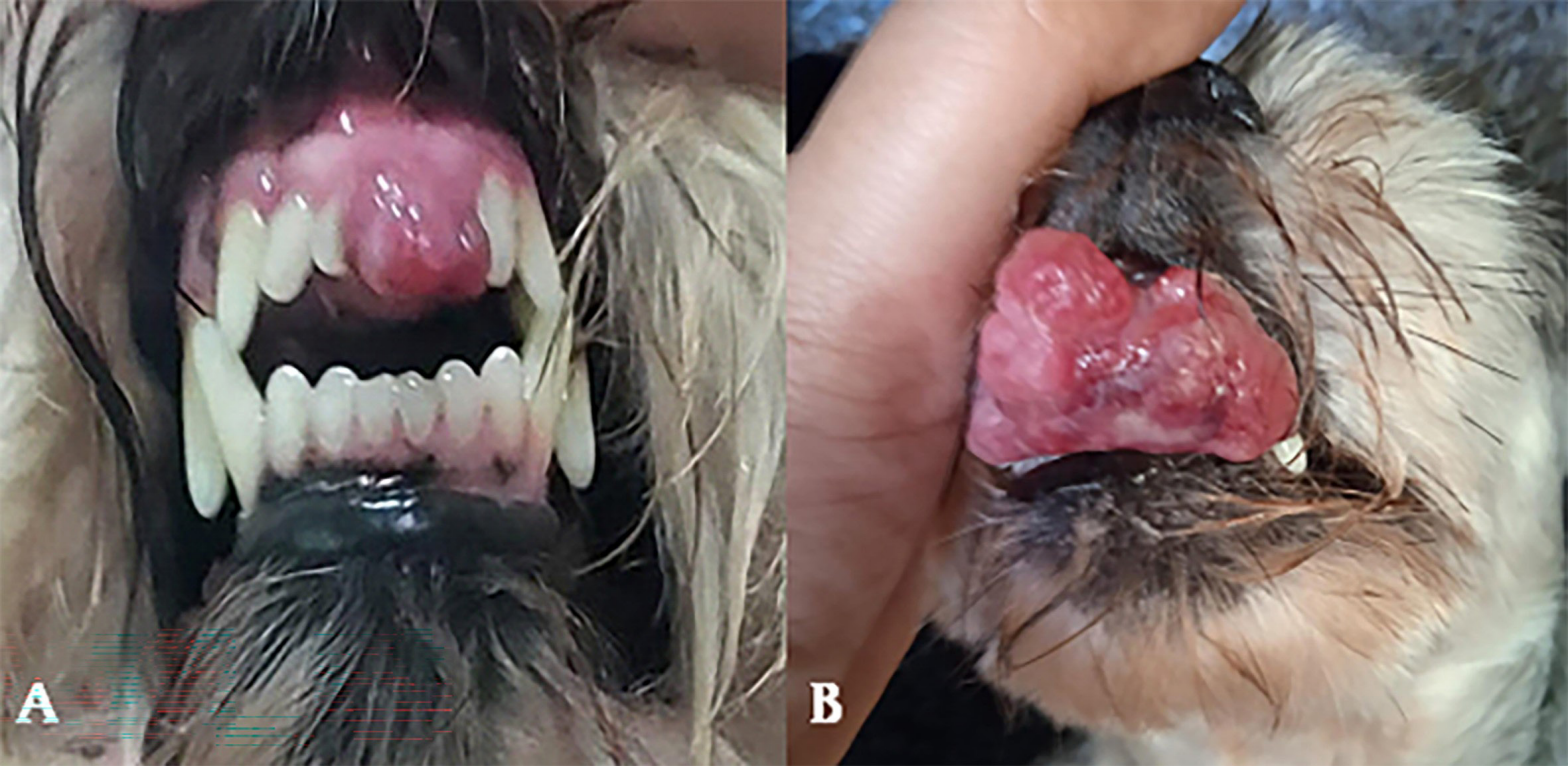
Clinical Presentation and Recurrence: (A) Rostral view of the nodule involving the incisive bone and upper incisor region at the time of initial marginal excision. (B) Local recurrence of the lesion observed five months after the primary surgical intervention.

A 10-year-old, 7 kg, castrated male Schnauzer was presented to a veterinary clinic in Conchal, São Paulo, with nodules involving the upper incisors (teeth 101, 102, 201, and 202) (Figure 1). The patient had a clinical history of progressive tumor growth in the incisor region for at least 30 days.

The initial complete blood count was within normal limits, though no skull radiographs were performed at that time. The nodules were surgically excised under inhalant anesthesia using an anesthetic protocol of acepromazine (0.02 mg/kg) and midazolam (0.2 mg/kg) for premedication, induction with propofol (4 mg/kg) and ketamine (1 mg/kg), and maintenance with isoflurane supplemented by a locoregional bupivacaine block.

Histopathological analysis revealed lobules of varying sizes and shapes organized around a central focus of bone, variably mineralized immature cartilage, or a combination of both. Surrounding this central matrix was a cuff of polygonal, hyperchromatic mesenchymal cells characterized by oval nuclei, condensed chromatin, and 1–2 prominent nucleoli. While discrete anisocytosis and anisokaryosis were noted, there was no evidence of necrosis or vascular invasion. A mitotic index of 2 mitotic figures per 10 high-power fields (FN22/40x; 2.37 mm^2^) confirmed a diagnosis of well-differentiated Multilobular Bone Tumor (MLTB), Grade I.

Despite the initial surgery, the tumor recurred five months after the marginal excision^7^. The patient presented with a 3.3 x 2.4 cm nodule invading the entire upper incisor region (Figure 1B), accompanied by halitosis and difficulty eating, which led to a significant deterioration of the animal's overall clinical condition.

The patient was referred for specialized oncological care, where a comprehensive workup was performed, including a complete blood count (CBC) and renal and hepatic biochemical profiles, prior to head computed tomography (CT).

The complete blood count (CBC) revealed a normocytic, normochromic anemia with erythrocytes at 5,53 × 10^6^/mm^3^ (Reference Interval [RI]: 5,7 a 7,4 x 10^6^/mm3), a mean corpuscular volume (MCV) of 69.44 fL (RI: RI: 63-77 fL), and a mean corpuscular hemoglobin concentration (MCHC) of 32.81% (RI: 21-35%). There was also leukocytosis with neutrophilia, characterized by a white blood cell count of 18x10^6^/ mm3 (RI: 6-16 x10^6^/mm^3^) and segmented neutrophils at 14.760 /µL (RI: 3,300-12,800/µL), without a left shift or evidence of hemoparasites. Biochemical evaluations of alanine aminotransferase, alkaline phosphatase, creatinine, and urea showed no significant abnormalities.

**Figure 2:**
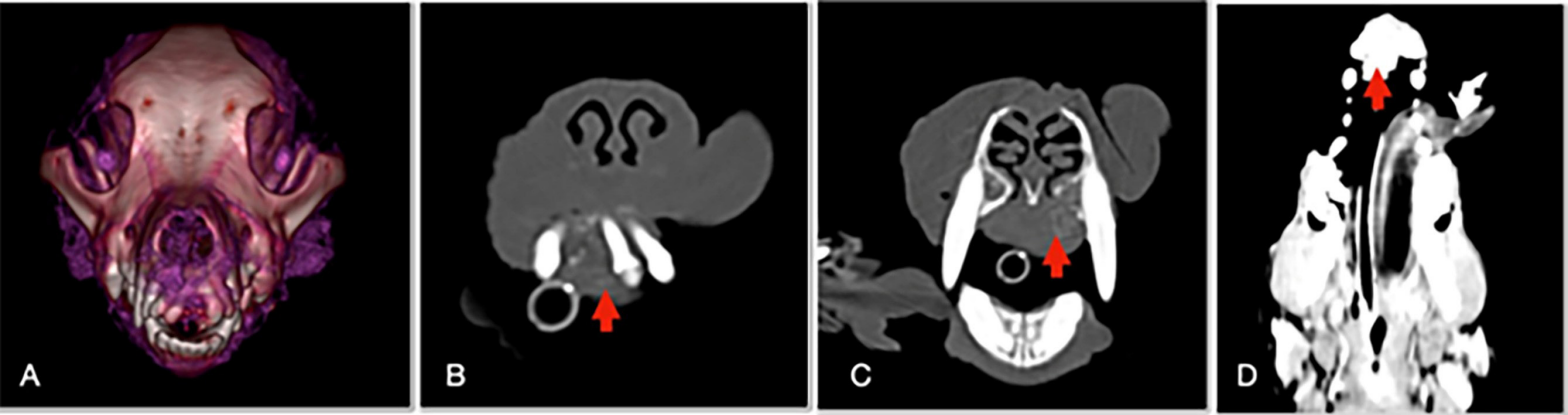
Computed Tomography (CT) Evaluation: 3D reconstruction (A), transverse (B and C), and axial (D) CT images displaying signs indicative of a neoplastic process involving the incisive bone at the level of the incisor teeth (red arrows). Images courtesy of CEDIVET – Veterinary Diagnostic Center.

Computed tomography (CT) findings identified a predominantly lytic, mixed bone reaction involving the incisive bone at the level of the incisor teeth. This was associated with a moderate increase in volume and subtle (discreet) contrast-enhancing heterogeneity within the regional soft tissues. The lesion measured approximately 3.1 x 2.3 x 1.6 cm (rostrocaudal x laterolateral x dorsoventral), indicative of a neoplastic process (Figure 2). Additionally, moderate periodontal disease was noted in the left upper and bilateral lower molars, alongside a mild (discreet) enlargement of the left mandibular and retropharyngeal lymph nodes. No abnormalities were detected in the nasal cavity or frontal sinuses.

A rostral maxillectomy was proposed to the owner; however, the procedure was not authorized. Consequently, electrochemotherapy (ECT) combined with tumor debulking was selected as an alternative therapeutic approach. The ECT was performed in accordance with recommended veterinary guidelines (Tellado et al., 2022). Bleomycin was administered as an intravenous bolus at a dose of 15,000 UI/m. Eight minutes following the injection, sequences of eight biphasic pulses were delivered using a clinical electroporator certified for veterinary use (Onkodisruptor; Biopulse Srl, Rome, Italy). These pulses utilized a voltage of 1000V/cm at a frequency of 1 Hz, with a duration of 50 + 50 µs per pulse and a 1 ms interval between pulses.

**Figure 3:**
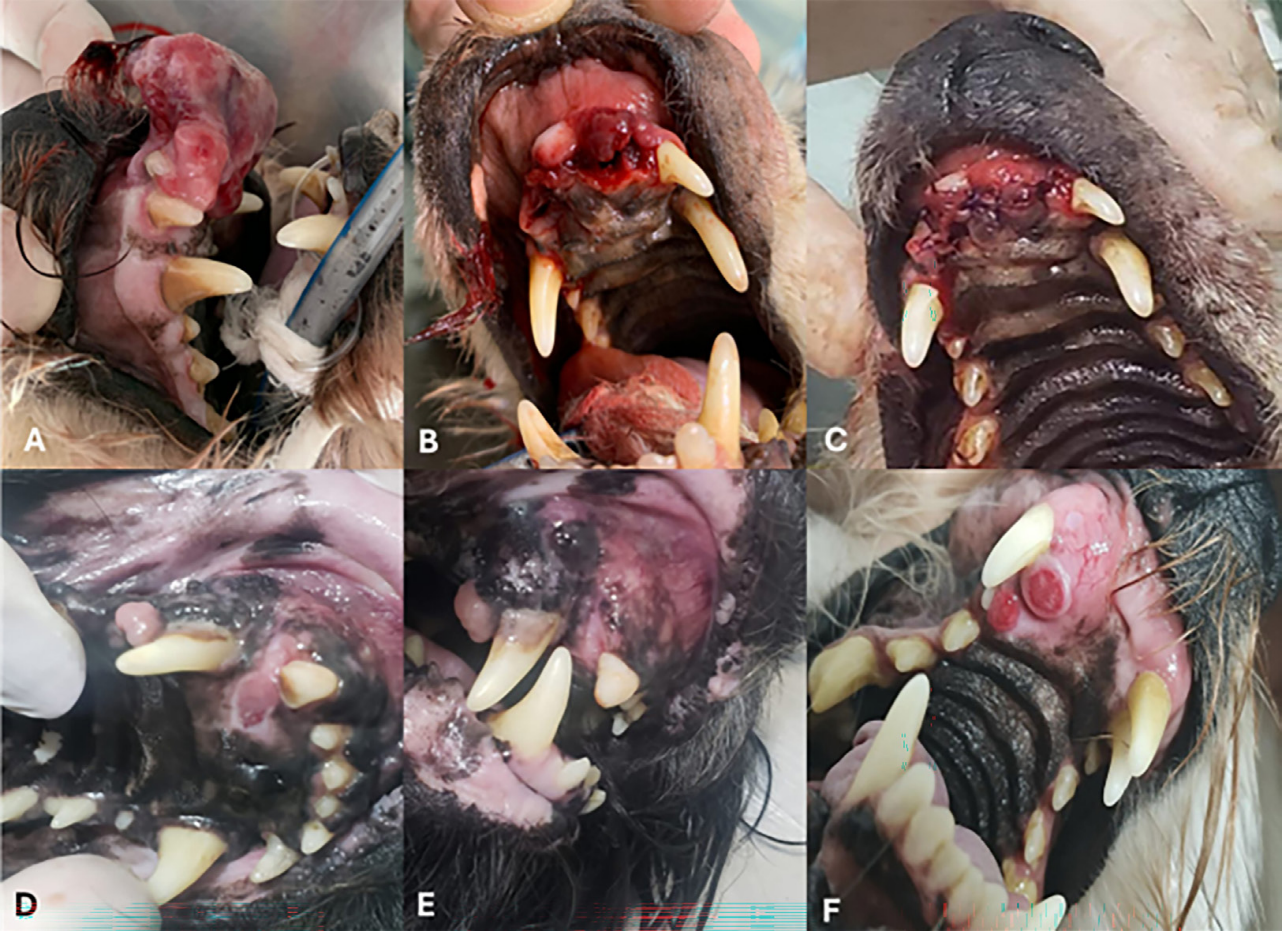
Intraoperative Procedure and Clinical Response: (A) Intraoperative view during electrochemotherapy (ECT) application. (B) Post-ECT appearance immediately following tumor debulking, showing a reduction in neoplastic tissue. (C) Surgical closure using 4-0 poliglecaprone absorbable monofilament suture. (D) Clinical appearance 28 days post-ECT. (E) Macroscopic complete remission (CR) as observed on a visual scale, with no detectable neoplastic tissue. (F) Local tumor recurrence in the palatal region adjacent to the right upper canine tooth (104), identified 435 days after the initial ECT procedure.

The surgical wound was closed using an absorbable monofilament suture (4-0 poliglecaprone) (Figure 3). Lymphadenectomy was not performed, as the computed tomography (CT) results showed no pathological changes or contrast enhancement in the regional lymph nodes, indicating a low suspicion of metastatic involvement.

Treatment response was evaluated according to the following Veterinary Cooperative Oncology Group (VCOG) criteria: Complete Response (CR), Disappearance of all target lesions. Partial Response (PR), A reduction of ≥ 30% in the sum of the diameters of the target lesions. Stable Disease (SD), A reduction of < 30% or an increase of < 20% in the sum of the diameters of the target lesions. Progressive Disease (PD): Development of new lesions or an increase of ≥ 20% in the sum of the diameters of the target lesions (Nguyen et al., 2015).

**Figure 4:**
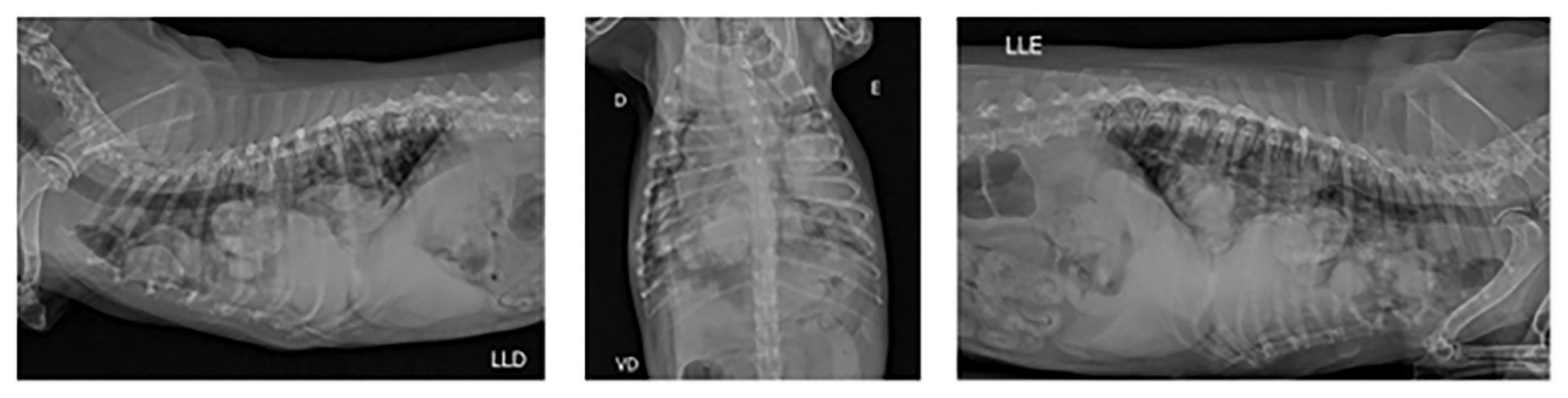
Metastatic Progression: Thoracic radiographs in right lateral (LLD), ventrodorsal (VD), and left lateral (LLE) projections. Findings include diffuse, variably sized pulmonary nodules throughout all lung lobes, consistent with advanced metastatic disease. Images courtesy of CEDIVET – Veterinary Diagnostic Center.

The patient achieved complete remission (CR) of the lesion, which was accompanied by normalized appetite, the absence of halitosis, and the resolution of eating difficulties. During follow-up examinations conducted every three months, no macroscopic alterations were observed in the upper incisor region, and the patient maintained a positive behavioral status. However, at 435 days post-ECT, local tumor recurrence was identified, alongside clinical signs of weight loss and hyporexia. Physical examination at that time revealed respiratory distress, and pulmonary metastasis was confirmed via three-view thoracic radiography. (Figure 4). The radiographs showed numerous nodular structures of soft-tissue radiopacity of varying dimensions, diffusely distributed throughout all lung fields.

Palliative chemotherapy with carboplatin was initiated at a dose of 10 mg/kg, administered intravenously over 10 minutes every 21 days. However, following the second treatment cycle, the patient’s respiratory condition deteriorated. Prednisone was subsequently prescribed for daily continuous administration. Due to progressive clinical deterioration and a poor prognosis, the patient was humanely euthanized, resulting in an overall survival time of 523 days from the initial diagnosis.

## Discussion

To the best of our knowledge, this is the first case report describing the treatment of canine MLTB with electrochemotherapy (ECT). The results demonstrate local efficacy and long-term disease control despite the tumor's infiltrative biological behavior. The oral cavity is a common site for neoplastic development, with oral tumors accounting for approximately 6% of all canine cancers. Literature suggests that 37% of oral cavity biopsies are malignant, while 34% are odontogenic. The most frequently diagnosed oral tumors include oral malignant melanoma (18.8–39.7%), squamous cell carcinoma (13.9–15%), fibrosarcoma (6.73–11.2%), and canine acanthomatous ameloblastoma (3.8–9.7%) (Tipirneni et al., 2024). In contrast, MLTB is considered a rare tumor with limited prevalence data. For instance, only 32 cases of MLTB in dogs were recorded over 10 years in the database of the Department of Pathology at the University of California, Davis. (Murphy et al., 2020).

The present report describes a 10-year-old Schnauzer, corroborating previous studies (Leonardi et al., 2014; Murphy et al., 2020), which indicate MLTB most commonly affects middle-aged dogs of medium to large breeds. A study by Dernell et al. (1998) involving 39 dogs with MLTB reported a mean age of 8 years, with no apparent breed or sex predilection. The clinical findings and tumor location in this case were consistent with literature descriptions, where MLTB typically presents as a firm, adherent mass arising from the bone surface, most frequently involving the maxillary and incisive bones (Leonardi et al., 2014; Murphy et al., 2020).

On radiographic evaluation, MLTB typically manifests as an expansive lesion containing punctate or nodular mineralized densities, which creates a characteristic "popcorn ball" appearance accompanied by adjacent osteolysis. Macroscopic examination generally reveals a firm nodular formation with a gritty consistency, ranging in color from gray to white or yellowish, occasionally exhibiting foci of necrosis.

Histologically, the tumor is characterized by a multilobular architecture where lobulations are separated by bundles of fibrous connective tissue. It features a classic trilaminar appearance and is composed of a central region of mineralized cartilage or osseous tissue. An intermediate zone is formed by fusiform to ovoid cells with a peripheral zone composed of fibrous tissue.

Although the mass is closely associated with bone margins, the perilesional connective tissue is typically displaced or compressed rather than infiltrated (Fontes et al., 2023). Furthermore, these tumors generally do not invade adjacent tissues and maintain a low mitotic index, often fewer than one mitotic cell per high-power field (HPF) (Psychas et al., 2009). In the present case, histopathology revealed lobules organized around a central focus of bone or mineralized immature cartilage (or sometimes a mixture of both), an absence of vascular invasion, and a low mitotic index of two mitotic figures in 10 HPF (22/40x), consistent with the characteristics of a Grade I MLTB.

Tumor classification is essential for determining a prognosis, as it serves as a critical predictor of biological behavior in dogs. A proposed grading system based on specific histological criteria and tumor invasiveness indicates that Grade III tumors are associated with shorter median times to local recurrence, metastasis, and overall survival compared to Grades I and II, although significant overlap exists within these ranges (Dernell et al., 1998).

Multilobular bone tumors (MLTB) are classified into three grades based on several critical histological criteria that determine their biological behavior. These factors include the nature of tissue interaction, specifically whether the tumor demonstrates compression versus active invasion of adjacent structures, as well as lobule characteristics such as size (small to medium vs. large) and the degree of organization (good, moderate, or poor). Additionally, mitotic activity is measured by the number of mitotic figures (1–5, 6–10, or >10), while cellular morphology is assessed by the degree of pleomorphism, ranging from monomorphic to marked. Finally, the presence or absence of necrosis is evaluated, with more pronounced histological changes indicating a higher level of malignancy and a more advanced classification (Murphy et al., 2020).

Research by Dernell et al. (1998) demonstrated that higher histological grades in canine multilobular bone tumors directly correspond to shorter intervals for recurrence, metastasis, and survival. For patients classified with Grade I tumors, the median disease-free interval was the longest at 1,332 days, compared to 782 days for Grade II and 288 days for Grade III. Similarly, the time to metastasis was significantly longer for Grade I (>820 days) than for Grade II (405 days) and Grade III (321 days). This trend continued in overall survival times, with Grade I patients surviving more than 897 days, while Grade II and Grade III patients had median survival times of 520 days and 405 days, respectively (Dernell et al., 1998).

Skull CT is an essential tool for investigating focal calvarial enlargement, particularly in cases where standard radiographs are inconclusive. This imaging modality allows for a significantly more detailed characterization of the lesion and its relationship with adjacent structures. Consequently, CT is highly effective for evaluating the precise extent of the lesion, which is critical for accurate surgical planning (Hathcock & Newton, 2000; Banks & Straw, 2004).

According to Hathcock and Newton (2000), who evaluated nine dogs with MLTB, the typical CT finding for these tumors is a radiopaque mass with a finely granular or punctate pattern and heterogeneous distribution, often affecting the caudal skull and invading the calvarium. Lesions originating from the zygomatic arch or the rostral cranial region may exhibit an even more granular internal texture, characteristics that become more evident when evaluated using specific bone windows. In the present case, however, the CT showed a predominantly lytic, mixed bone reaction involving the incisive bone, accompanied by a moderate increase in volume and discreetly contrast-enhancing heterogeneity with poorly defined soft tissue limits. Notably, this finding did not present the characteristic "popcorn" appearance or the finely granular radiopaque pattern typically reported in the literature (Vedrine, 2023).

While surgical removal with wide safety margins remains the most effective strategy for treating MLTB, up to 37% of patients may experience complications, with 38% of those cases being classified as severe. Mandibulectomy and maxillectomy are standard recommendations for oral tumors, yet they carry risks of complications such as hemorrhage, aspiration pneumonia, dehiscence, malocclusion, and oronasal fistula. Procedures located more caudally are associated with an even higher risk of severe hemorrhage and the potential need for blood transfusions (Cray et al., 2021). Furthermore, in advanced stages of the disease, surgery alone or combined with adjuvant therapies like radiotherapy and chemotherapy rarely achieves lasting remission (Tipirneni et al., 2024).

According to Dernell et al. (1998), patients undergoing surgery with compromised margins experience local recurrence significantly sooner, with a mean duration of 320 days. In contrast, patients who undergo surgery with clean margins achieve a much longer mean disease-free interval of 1,332 days. Additionally, the median survival time for patients with mandibular tumors is 1,487 days, compared with 528 days for tumors in other locations, such as the maxilla, orbit, and calvarium. This discrepancy partly reflects the lower capacity for achieving adequate surgical resection in maxillary neoplasms compared to mandibular neoplasms.

Recurrence of this neoplasm typically manifests after a prolonged period, with mean intervals ranging from 288 to 1,332 days depending on the tumor grade. This results in a relatively long disease-free interval and extended survival for dogs that undergo surgical treatment (Cook et al., 2017). However, according to the literature, more than 50% of affected dogs eventually develop metastasis, with the lungs identified as the most common site of dissemination (Dernell et al., 1998).

The median survival time for dogs treated with surgery, with or without adjuvant chemotherapy or radiotherapy, is approximately 22 months, with a mean time to local recurrence of 14 months. In stark contrast, the survival of affected dogs receiving chemotherapy alone is significantly shorter, with a reported mean of only 144 days (Cook et al., 2017).

Hypofractionated radiotherapy can be employed in cases of MLTB to prolong disease-free intervals. According to Sritrakoon et al. (2023), hypofractionated radiotherapy combined with partial orbitectomy in a dog with MLTB resulted in no local recurrence or distant metastasis for a period of one year and eight months, suggesting its potential to extend the disease-free interval.

Another therapy described in the literature for treating MLTB with compromised surgical margins is imatinib, a tyrosine kinase inhibitor. Reports indicate that this molecule is also effective against other neoplasms, including mast cell tumors, gastrointestinal stromal tumors (GIST), and sarcomas. Because tyrosine kinase receptors play a vital role in regulating intercellular communication and controlling cell growth, proliferation, differentiation, and metabolic survival, their inactivation may assist in controlling tumor growth. According to the authors, this therapy can be utilized in cases where wide resection is unfeasible and the tumor expresses these receptors, thereby improving clinical signs by decelerating tumor progression (Lee et al., 2022).

Electrochemotherapy (ECT) has been extensively researched in human medicine for managing oral, head, and neck tumors. In veterinary medicine, several studies are currently investigating the treatment of canine oral tumors, with a primary focus on melanoma and squamous cell carcinoma. ECT serves as a viable option for treating inoperable tumors and has demonstrated effectiveness as a standalone therapy. Notably, in cases where only a partial response or treatment failure occurs, the procedure can be repeated, often maintaining equal or even superior efficacy compared to the initial applications (Tozon et al., 2016).

The use of electrochemotherapy (ECT) combined with the administration of chemotherapeutic agents, such as cisplatin, bleomycin, or a combination of both, has been documented in veterinary literature (Moretti et al., 2022). Clinical efficacy is typically inversely proportional to tumor size; specifically, tumors smaller than 2 cm^3^ demonstrate a better response rate compared to larger masses (Nemec et al., 2020). Furthermore, the application of intravenous bleomycin and ECT following surgical debulking has shown promising results in other bone-related cancers; for instance, a case of canine osteosarcoma managed with this combined approach resulted in successful tumor control for 16 months (Taques et al., 2021).

Literature on the use of electrochemotherapy (ECT) in cases of multilobular bone tumor (MLTB) is scarce. Most reports concerning ECT in oral neoplasms focus on oral melanoma due to its higher incidence in dogs. A study by Moretti et al. (2022) evaluated the use of ECT in 19 dogs with various oral neoplasms, including 13 with melanoma, two with squamous cell carcinoma (SCC), two with sarcomas, and one with lymphoma. In that study, the median disease-free interval was 180 days for melanoma, 225 days for SCC, and ranged between 75 and 300 days for sarcomas and was 300 days for lymphoma.

While the sample size for oral sarcomas in the literature is not yet representative, it serves as a valuable basis for comparison. In the present case, the use of ECT for MLTB resulted in a disease-free interval superior to those reported for other oral sarcomas. This suggests that ECT may be considered a viable alternative treatment for tumors that cannot be completely excised, particularly in cases where owners refuse extensive or radical surgery.

## Conclusion

In this single-case report, electrochemotherapy (ECT) demonstrated a satisfactory therapeutic response as an alternative to radical surgical procedures, providing effective local control and a disease-free interval of 435 days (extending beyond the 365-day mark). These findings open new possibilities for managing multilobular bone tumors (MLTB) in patients who are not candidates for surgery or in instances where the owner declines invasive surgical options. Ultimately, ECT proved to be a safe and effective technique with minimal adverse effects for this patient.
